# Gall-specific promoter, an alternative to the constitutive *CaMV35S* promoter, drives host-derived RNA interference targeting *Mi-msp2* gene to confer effective nematode resistance

**DOI:** 10.3389/fpls.2022.1007322

**Published:** 2022-11-08

**Authors:** Ila Joshi, Anil Kumar, Deshika Kohli, Ramcharan Bhattacharya, Anil Sirohi, Ashok Chaudhury, Pradeep K. Jain

**Affiliations:** ^1^ ICAR-National Institute for Plant Biotechnology, New Delhi, India; ^2^ Department of Bio and Nano Technology, Bio & Nano Technology Centre, Guru Jambheshwar University of Science and Technology, Hisar, Haryana, India; ^3^ Department of Entomology, Nematology and Chemistry Units, Agricultural Research Organization, The Volcani Center, Bet Dagan, Israel; ^4^ Division of Nematology, ICAR-Indian Agricultural Research Institute, New Delhi, India

**Keywords:** HD-RNAi, *Meloidogyne incognita*, p*At2g18140*, gall specific promoter, *Mi-msp2*, effector gene

## Abstract

One of the major obligate plant parasites causing massive economic crop losses belongs to the class of root-knot nematodes (RKNs). Targeting of major nematode parasitism genes *via* Host Delivered-RNAi (HD-RNAi) to confer silencing is established as one of the most effective approaches to curb nematode infection. Utilizing nematode-responsive root-specific (NRRS) promoters to design a dsRNA molecule targeting approach to hamper nematode parasitism. Here, a previously validated peroxidase gall specific promoter, p*At2g18140*, from *Arabidopsis* was employed to express the dsRNA construct of the nematode effector gene *Mi-msp2* from *Meloidogyne incognita*. *Arabidopsis* RNAi lines of *CaMV35S::Mi-msp2-RNAi* and p*At2g18140::Mi-msp2-RNAi* were compared with control plants to assess the decrease in plant nematode infection. When subjected to infection, the maximum reductions in the numbers of galls, females and egg masses in the *CaMV35S::Mi-msp2-RNAi* lines were 61%, 66% and 95%, respectively, whereas for the p*At2g18140::Mi-msp2-RNAi* lines, they were 63%, 68% and 100%, respectively. The reduction in transcript level ranged from 79%-82% for *CaMV35S::Mi-msp2-RNAi* and 72%-79% for the p*At2g18140::Mi-msp2-RNAi* lines. Additionally, a reduction in female size and a subsequent reduction in next-generation fecundity demonstrate the efficacy and potential of the gall specific promoter p*At2g18140* for utilization in the development of HD-RNAi constructs against RKN, as an excellent alternative to the *CaMV35S* promoter.

## 1 Highlights

- Reduced nematode infection in RNAi lines *CaMV35S::Mi-msp2-RNAi* and *pAt2g18140::Mi-msp2-RNAi.*
- Reduced fecundity and size of females in RNAi lines for both promoters.
- The gall-specific promoter is very effective in gene silencing *via* HD-RNAi.

## 2 Introduction

Plant parasitic nematodes (PPNs) have been identified as one of the major plant pest groups causing large crop losses annually (Chitwood, 2003). The major groups among the PPNs are root knot nematodes (RKN- *Meloidogyne* spp.) and cyst nematodes (*Heterodera* spp. and *Globodera* spp.). Among the RKNs, *Meloidogyne incognita* (southern root knot nematode) is a sedentary obligate parasite with a dynamic geographical distribution (Abad et al., 2008). With annual crop losses of USD 173 billion and a wide host range of as many as 2000 crop plant species, RKN is one of the primary parasites threatening agriculture (Elling, 2013). The major feature that enables *M. incognita* to successfully parasitize host plants for a longer duration is the formation of permanent nematode feeding sites (NFSs) comprising giant cells. Infective juveniles (J2s) penetrate the host plant roots upon receiving a suitable stimulus in the soil and then migrate to the cortex region to develop giant cells (Jones and Payne, 1978).

The functional mechanisms for the establishment, construction and later maintenance of the NFS are strategically controlled by a group of nematode parasitism genes known as effector genes (Gheysen and Mitchum, 2011). The effector proteins encoded by the effector genes have been reported to play a vital role in nematode parasitism. 16D10, calreticulin and MSP18 nematode effector proteins are functionally involved in disrupting the host plant basal immune system and rendering the host plant prone to infection (Huang et al., 2006; Jaouannet et al., 2012; Jaouannet et al., 2013; Grossi-de-Sa et al., 2019). Effector protein 8D05 influences water transport to nematode feeding sites, and protein Mj-FAR-1 is involved in modifying cell wall-related plant gene functions for developing feeding sites (Iberkleid et al., 2013; Xue et al., 2013). However, the effector protein Mi-MSP40 is actively involved in suppressing the effector triggered immunity (ETI) signalling-associated cell death response in the host (Niu et al., 2016).

The availability of plant genome sequences has made it feasible to characterize the functional aspects of their genes, leading to immense progress in plant genome research. One of the most applicable tools for functional genomics studies is reverse genetics. In the field of reverse genetics, *in vivo* dsRNA delivery *via* Host Delivered-RNAi (HD-RNAi) technology has proven to be an effective method for gene silencing (Timmons et al., 1998). For the purpose of combating several PPNs, many effector genes have been targeted by HD-RNAi and successfully silenced. Silencing of various effector genes has directly affected the functionality of nematode parasitism to different degrees, as reported in the cases for different effectors genes termed as *Meloidogyne* secretory proteins of *M. incognita* (*Mi-msp*) (Joshi et al., 2022).

The majority of promoters used in gene silencing *via* RNAi are expressed constitutively, such as *CaMV35S* and *pUbi1* (Sindhu et al., 2009). Lacking restrictive spatial expression, the constitutive promoters drive the dsRNA expression in RNAi-based gene construct throughout the plant tissues/organs in transgenic plants. By silencing the target gene and its homologues in all of the plant tissues, the constitutive promoter poses the risk of causing off-target pleiotropic phenotypes thus altering the desired results (Peremarti et al., 2010). One of the major risks in such cases is embryo lethality caused by silencing of homologous sequences. Selective functionality of the common transgenic promoter, *CaMV35S*, for expression of nematode RNAi constructs in NFS is questionable (Goddijn et al., 1993; Bertioli et al., 1999). The *CaMV35S* driven *gusA* and *gfp* have been reported to be down-regulated at NFS of RKN and cyst nematodes in *Arabidopsis thaliana* (Urwin et al., 1997). Thus, due to a many off target effects across homologous sequences in different tissues/organs it is difficult to assess the reason for phenotypic variations in transgenic plants compared to the control ones. On the other hand employing regulated promoters proves as a better option for expression of RNAi constructs these regulated promoters are stage specific, expressed in certain selective tissues/organs, and can be physically/chemically induced by specific external or internal stimuli. The use of stress-inducible promoters is highly desirable due to a potential decrease in off target effects (Yamaguchi-Shinozaki and Shinozaki, 2001). Previously a range of NRRS promoters have been successfully identified and employed to combat PPN infection to varying degrees (Opperman et al., 1994; Thurau et al., 2003; Sukno et al., 2006; Kumar et al., 2016). The *A. thaliana* promoter *MDK4-20* and the *Zea mays* promoter *ZmRCP-1* are expressed specifically in the root tip making them suitable to control the expression of the antinematode defence genes and dsRNA molecules targeting PPN genes (Lilley et al., 2011; Onyango et al., 2016). Therefore, the study of RNAi constructs under the control of NRRS promoters will provide insights into the molecular functionality of the nematode responsive promoters as well as the mechanisms of their effector genes.

The gall-specific promoter p*At2g18140*, which drives the expression of endogenous peroxidase in A. thaliana, has been previously reported as responsive to infection by M. incognita. (Kakrana et al., 2017). A substantial reduction in the gene expression of the effector gene *Mi-msp2* was reported, which led to a decrease in parasitization by *M. incognita*. Also, there was evident reduction in infection in phenotype in of the *Mi-msp2* RNAi lines in comparison with the control plants (Joshi et al., 2019). The adult *M. incognita* females feeding on the *Mi-msp2* RNAi lines presented an up to 85% reduction in gene transcript levels (Joshi et al., 2019). On the other hand, a previously reported gall specific promoter, *At2g18140*, was found to express specifically at the sight of *M. incognita* infection in the transgenic lines (Kakrana et al., 2017). In light of these promising results in this study the RNAi construct was prepared expressing siRNA of the effector gene *Mi-msp2* under the control of the gall specific promoter *At2g18140*. A comparative study was performed to assess the reduction in *M. incognita* infestation to evaluate the efficacy levels of effector gene *Mi-msp2* silencing in the *Arabidopsis* HD-RNAi lines driven by the *CaMV35S* and *At2g18140* promoters. Five independent events of each *CaMV35S::Mi-msp2-RNAi Arabidopsis* line and p*At2g18140::Mi-msp2-RNAi Arabidopsis* line were evaluated along with control *Arabidopsis* (*Col-1*) plants.

## 3 Material and methods

### 3.1 Procurement and maintenance of the *M. incognita* culture on tomato plants (PUSA Ruby)

The Division of Nematology, Indian Agricultural Research Institute (IARI), New Delhi, provided the pure culture of RKN *M. incognita* (Kofoid & White) Chitwood race 1, which was maintained on Pusa Ruby tomato (*Solanum lycopersicum L.*) plants at ambient culture room conditions at NIPB-IARI. The identification of *M. incognita* was performed from an isolated single egg mass from a pure culture (Zijlstra et al., 2000; Joshi et al., 2019). Hatched J2s obtained from pure cultured egg masses were used for multiplication and culture maintenance. The host plant, PUSA Ruby tomato seedlings, was grown and irrigated with Hoagland solution at regular intervals (Joshi et al., 2019). Twenty-five-day-old plants were subjected to *M. incognita* juvenile (500) infection, and the infected roots were harvested and analysed for gall development after 6 to 7 weeks of infection. The plants were harvested after the developed egg masses were visible; the latter were then hand-picked with forceps. The isolated egg masses were allowed to hatch immersed in M9 medium at 28°C over a wire gauge covered with tissue paper on Petri plates (the modified Baermann funnel technique) (Baermann, 1917; Hooper, 1986). The released J2s were collected for the maintenance of a continuous culture of *M. incognita* and for transgenic infection analyses.

### 3.2 Preparation of RNAi *constructs pAt2g18140::Mi-msp2-RNAi* and *CaMV35S::Mi-msp2-RNAi*

#### 3.2.1 Cloning of sense, antisense and promoter fragments in the RNAi plasmid pHANNIBAL

**Figure 1:**
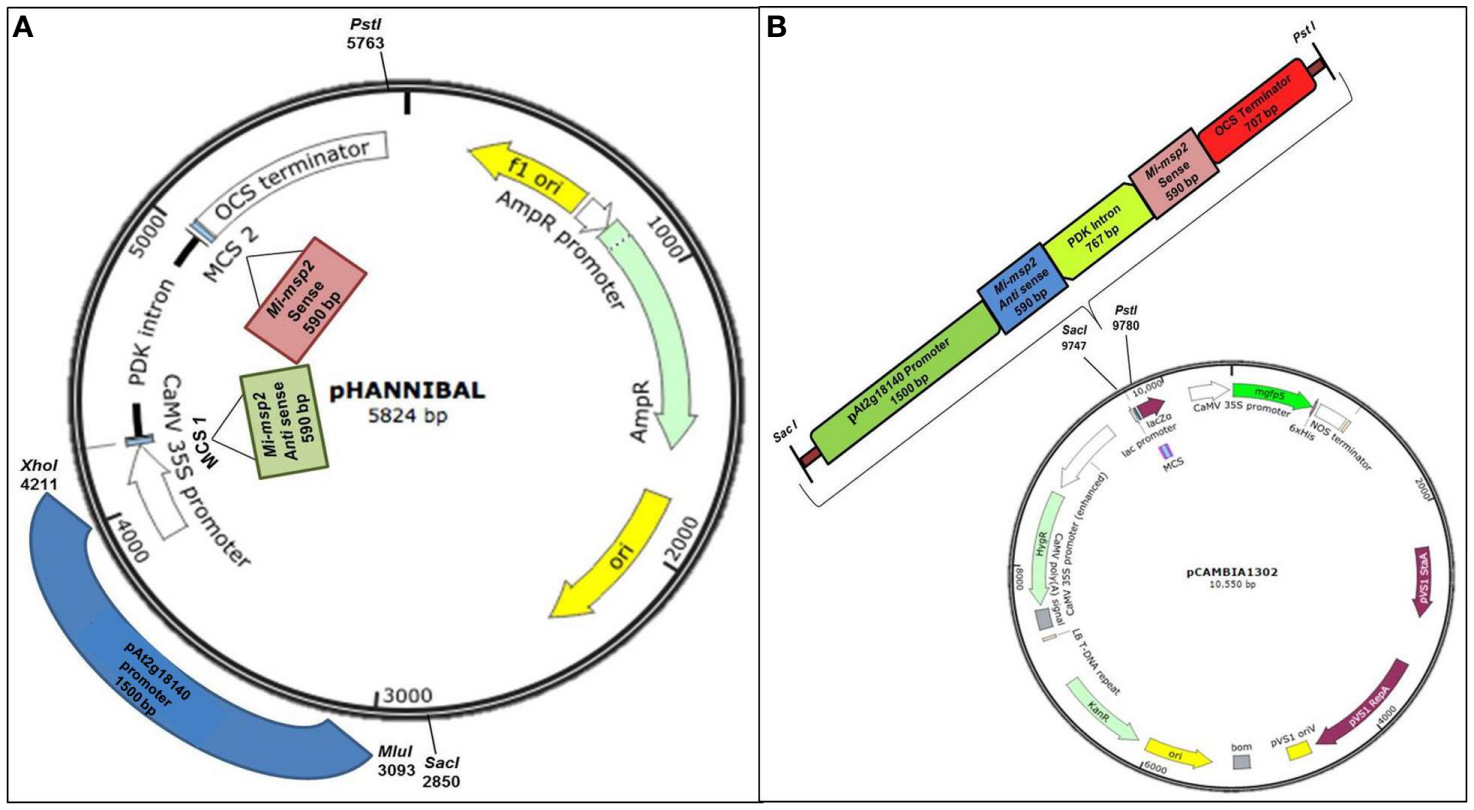
Constructs used in the study. **(A)** Cloning of the *Mi-msp2* gene and promoter p*At2g18140* in the RNAi vector p*HANNIBAL*. **(B)** Insertion of the RNAi cassette from p*HANNIBAL* into the binary vector p*CAMBIA 1302* expressing the dsRNA construct for p*At2g18140::Mi-msp2-RNAi*.

Selective primers for the sense and antisense sequences of the *Mi-msp2* gene and the gall specific promoter p*At2g18140* were synthesized with flanking sequences for suitable restriction enzymes using Primer3 plus (**Supplementary Table S1**). The gall specific promoter of the *At2g18140* gene (1572 bp) was amplified from DNA isolated from *Arabidopsis (Col-0)* plants using gene-specific primers and the *Mi-msp2* gene fragments (sense and antisense- 590 bp) were amplified from *M. incognita* DNA with gene-specific primers (**Supplementary Table S1**). Primers for cloning in p*HANNIBAL* (Wesley et al., 2001) were designed from the *Mi-msp2* gene plus restriction sites for *Eco*RI and *Kpn*I for the sense fragment and *Hind*III and *Xba*I for the antisense fragment. For the promoter gene *At2g18140*, primers with flanking sequences for the enzymes *Mlu*I and *Xho*I were designed suitably for cloning in p*HANNIBAL* (Wesley et al., 2001) (**Figure 1A**). The amplicons were purified *via* agarose gel electrophoresis, cloned into the p*GEMT* easy vector (Promega Corporation, Wisconsin-Madison, USA) and confirmed by Sanger sequencing. For cloning, the gene fragments were digested with suitable restriction enzymes from p*GEMT* plasmids and confirmed by sequencing. The digested fragments were further purified and ligated within the digested RNAi vector p*HANNIBAL* (**Figure 1A**). The ligated vector was transformed into *E. coli* (DH5α), and confirmation of the positive clones was performed by a colony PCR protocol (**Supplementary Table S1**). The positive clones were further selected for cloning the RNAi cassette into the binary vector p*CAMBIA 1302* for transformation into *A. thaliana* (*Col-0*) plants.

##### 3.2.1.1 RNAi cassette transfer from pHANNIBAL to the binary vector pCAMBIA 1302

The RNAi cassette from transformed p*HANNIBAL* was restricted with *Sac*I and *Pst*I and cloned into the digested binary vector p*CAMBIA 1302 via* ligation. The ligated vector was transformed into *E. coli* (DH5α) (**Figure 1B**). A colony PCR protocol was used to confirm transformed positive clones. The RNAi cassette bearing the p*CAMBIA 1302* plasmid was isolated and transformed into the *Agrobacterium tumefaciens* GV3101 strain by the standard freeze-thaw method (Clough and Bent, 1998). *A. tumefaciens* was grown on selective YEP medium (50 mg/L kanamycin, 50 mg/L gentamycin and 25 mg/L rifampicin) for the selection of recombinant transformants. The transformed *A. tumefaciens* GV1301 was confirmed to contain the insert by colony PCR. Wild-type *Arabidopsis thaliana* was transformed with positive *A. tumefaciens* clones *via Agrobacterium-*mediated transformation *via* floral dip method (Clough and Bent, 1998). The seeds harvested from the T_1_ plants were screened on selective MS media (15 mg/L hygromycin), and the selected transgenic p*At2g18140::Mi-msp2-RNAi* plants were confirmed by PCR (**Figure S2**). The T_1_ transgenic p*At2g18140::Mi-msp2-RNAi* plants were propagated up to the T_3_ homozygous generation (**Figure S1**).

#### 3.2.2 Transformation of *Arabidopsis thaliana* (Col-1) *with the CaMV35S::Mi-msp2-RNAi* vector pYSB

The *CaMV35S::Mi-msp2-RNAi* construct was prepared using Gateway technology (Invitrogen Corpration, Waltham, Massachusets, USA) in the RNAi plant expression vector p*YSB* (generated by IIT Kanpur). The recombinant p*YSB* RNAi construct was transferred into *Agrobacterium tumefaciens* strain GV3101 and introduced into *Arabidopsis thaliana* (*Col-1*) *via Agrobacterium-*mediated transformation (Clough and Bent, 1998). T_3_ homozygous *CaMV35S::Mi-msp2-RNAi* transgenic lines were used for this study (Joshi et al., 2019).

### 3.3 Seed storage and sterilization for plating on MS media

The seeds of *A. thaliana* (*Col-1*) and five independent events of each *CaMV35S::Mi-msp2-RNAi* line and p*At2g18140::Mi-msp2-RNAi* line were taken (approximately 100–150 seeds each) for sterilization and plating. The seeds were placed in a microfuge tube and surface sterilized by the standard two-step protocol (Joshi et al., 2019). The sterilized seeds were suspended in a 0.1% agarose solution. These seeds were then kept at 4°C for 72 hours for vernalization and plated on selective MS media (pH 5.8) (HiMedia) with the antibiotic hygromycin (15 mg/ml) for p*At2g18140::Mi-msp2-RNAi* lines and kanamycin (50 mg/L) for the *CaMV35S::Mi-msp2-RNAi* lines. Within 12–15 days, the resistant plants grew with bright green and healthy leaves and a well-developed root system after plating and they were allowed to grow until the 6-leaf stage. The plants were grown under a 16-h light/8-h dark photoperiod at 21°C. After 14 days, the seedlings (ten plants per plate) were transferred to half concentration MS media with CleriGel (HiMedia) in round Petri dishes (9 cm diameter). The plants were kept slightly angled at 45° for unidirectional root proliferation for 7 days until secondary roots appeared. The *A. thaliana* (*Col-1*), p*At2g18140::Mi-msp2-RNAi* lines and *CaMV35S::Mi-msp2-RNAi* lines showed no differences in their morphological features, such as root and shoot growth patterns, life cycle or flowering responses. Furthermore, no phenotypic distinctions were observed among the different RNAi lines.

### 3.4 Nematode J2 sterilization, infection of the plants and analyses of the infected plants

*M. incognita* J2s obtained after hatching at 28°C were suspended in M9 buffer and sterilized by the standard protocol using nematode sterilization buffer (Kakrana et al., 2017). After secondary root growth, 21-day-old plants were infected with approximately 300 *M. incognita* J2s per plant. Thirty-five days post infection (dpi), the plants were carefully harvested from the MS media (CleriGel), and the fresh weight of the root mass was recorded. The roots were visualized under a light microscope to count the numbers of galls, females and egg masses per gram of root fresh weight. The egg masses were picked with forceps, and the females were extracted after dissecting the roots under a light microscope. A mean of 10–15 biological replicates was taken for the counted numbers of galls, females and egg masses per gram of root fresh weight per RNAi line. The data obtained from the control plants versus the p*At2g18140::Mi-msp2-RNAi* and *CaMV35S::Mi-msp2-RNAi* RNAi lines were compared to assess the gene-silencing efficiencies.

### 3.5 Reproductive capacity and fecundity of M. incognita in the *A. thaliana (Col-1), pAt2g18140::Mi-msp2-RNAi* lines and the *CaMV35S::Mi-msp2-RNAi* lines

To estimate the level of susceptibility of the *CaMV35S::Mi-msp2-RNAi* and p*At2g18140::Mi-msp2-RNAi* line plants, the *M. incognita* egg masses were isolated, and the number of eggs in each single egg mass was recorded. Five egg masses each were isolated from the five independent *CaMV35S::Mi-msp2-RNAi* lines (*CaMV35S::Mi-msp2-RNAi* E1, *CaMV35S::Mi-msp2-RNAi* E2, *CaMV35S::Mi-msp2-RNAi* E3, *CaMV35S::Mi-msp2-RNAi* E4 and *CaMV35S::Mi-msp2-RNAi* E5) and from the five independent p*At2g18140::Mi-msp2-RNAi* lines (p*At2g18140::Mi-msp2-RNAi* E1, p*At2g18140::Mi-msp2-RNAi* E2, p*At2g18140::Mi-msp2-RNAi* E3, p*At2g18140::Mi-msp2-RNAi* E4 and p*At2g18140::Mi-msp2-RNAi* E5). The isolated egg masses were kept for hatching at 28°C, and the number of J2s that hatched was recorded. An average of the total number of eggs was calculated for *CaMV35S*::*Mi-*msp2, p*At2g18140::Mi-msp2-RNAi* and the control plants from the respective isolated egg masses. A comparative evaluation of the averaged values of the egg masses was made for the *CaMV35S::Mi-msp2-RNAi* lines, p*At2g18140::Mi-msp2-RNAi* lines and the control plants. The susceptibility of the control plants versus the p*At2g18140::Mi-msp2-RNAi* and *CaMV35S::Mi-msp2-RNAi* lines after *Mi-msp2* gene silencing was assessed by Oostenbrink’s reproduction factor (Oostenbrink, 1966; Joshi et al., 2019). Phenotypic effects on the infected roots were acquired at a suitable magnification with a NIKON^®^ microscope. The extracted females were also captured to compare the sizes (lengths and widths) of 20 nematode females (NIS element D- Nikon Instruments Inc., New York, USA). The average values were taken for further analyses.

### 3.6 Comparative gene expression analysis in RNAi females after gene silencing

The adult feeding females were carefully excised from control plants and five events of each p*At2g18140::Mi-msp2-RNAi* and *CaMV35S::Mi-msp2-RNAi* lines. The excised females (200-250 per sample) were flash-frozen in liquid nitrogen for total RNA isolation (PureLink RNA Mini Kit-Thermo Fisher Scientific, Wilmington, USA). Around 250-300 ng/μl RNA was isolated from each sample. For expression analysis by qRT-PCR (Applied Biosystems StepOne Plus™ Real-Time PCR (USA, Massachusetts), cDNA was synthesized from the total RNA (SuperScript III First-Strand Synthesis System-Thermo Fisher Scientific, Wilmington, USA). RT primers designed for the *Mi-msp2* cDNA sequence were used for the expression analysis (http://frodo.wi.mit.edu/primer3/) (**Supplementary Table S1**). Quantitative real-time PCR was performed to analyse the gene expression of the *Mi-msp2* gene in females feeding on each of the five RNAi lines of *CaMV35S::Mi-msp2-RNAi* and p*At2g18140::Mi-msp2-RNAi*, each expressing dsRNA specific for the *Mi-msp2* gene. The females feeding on control plants were also isolated and analysed for *Mi-msp2* gene expression. The ΔΔCt values of the obtained gene expression of the effector gene *Mi-msp2* were compared for females feeding on the control plants, and five events for each p*At2g18140::Mi-msp2-RNAi* and *CaMV35S::Mi-msp2-RNAi* line were evaluated using nematode *actin* reference gene (Accession no. *Mi-actin*- Minc06769) (Livak and Schmittgen, 2001). For each event three biological and three technical replicates were taken. For the assessment of significance, statistical analysis using one-way ANOVA and Tukey’s test were performed.

## 4 Results

### 4.1 Phenotypic analyses of control and transgenic lines before and after *M. incognita* infection

All of the transgenic lines of both RNAi constructs (*CaMV35S::Mi-msp2-RNAi* and p*At2g18140::Mi-msp2-RNAi)* were plated on selective MS media and observed for phenotypic differences in plant growth. Twelve-day-old seedlings of transgenic lines were found to have similar growth patterns to that of the control plants (wild-type *Col-0*). There were no phenotypic differences observed among the different RNAi lines.

### 4.2 *M. incognita* infection assay on *Mi-msp2 HD-RNAi* lines of *Arabidopsis thaliana*

**Figure 2:**
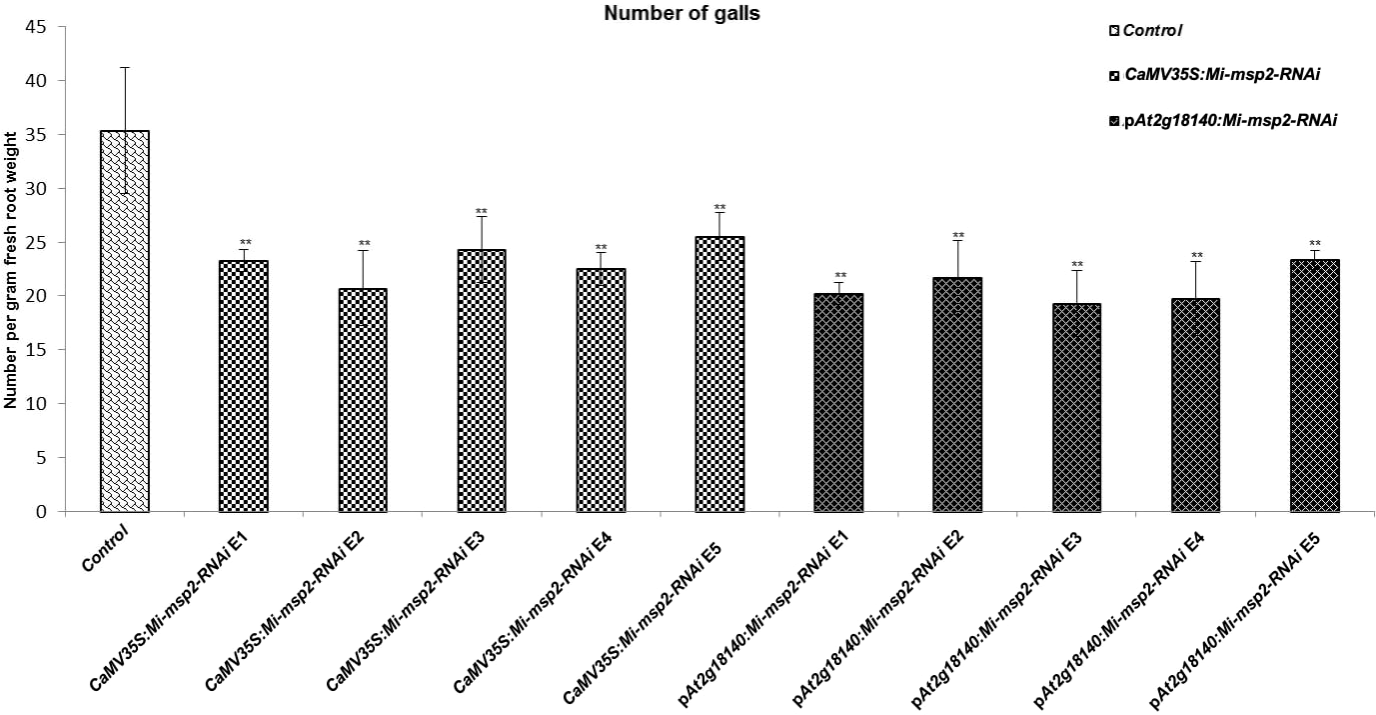
Five independent RNAi lines each for constructs *CaMV35S::Mi-msp2-RNAi* and p*At2g18140::Mi-msp2-RNAi* exhibit a reduction in infection represented by a reduction in the number of galls compared to the control line evaluated for *M. incognita* infection. Each bar represents the mean ± SE. Double asterisks indicate statistically significant differences by one-way ANOVA and Tukey’s test (p ≤ 0.01).

**Figure 3:**
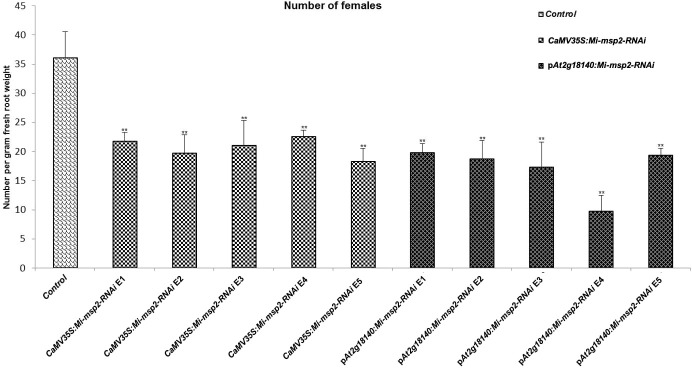
Five independent RNAi lines each for constructs *CaMV35S:Mi-msp2* and p*At2g18140::Mi-msp2-RNAi* exhibit a reduction in infection represented by a reduction in the number of females isolated from infected roots compared to the control line evaluated for *M. incognita* infection. Each bar represents the mean ± SE. Double asterisks indicate statistically significant differences by one-way ANOVA and Tukey’s test (p ≤ 0.01).

**Figure 4:**
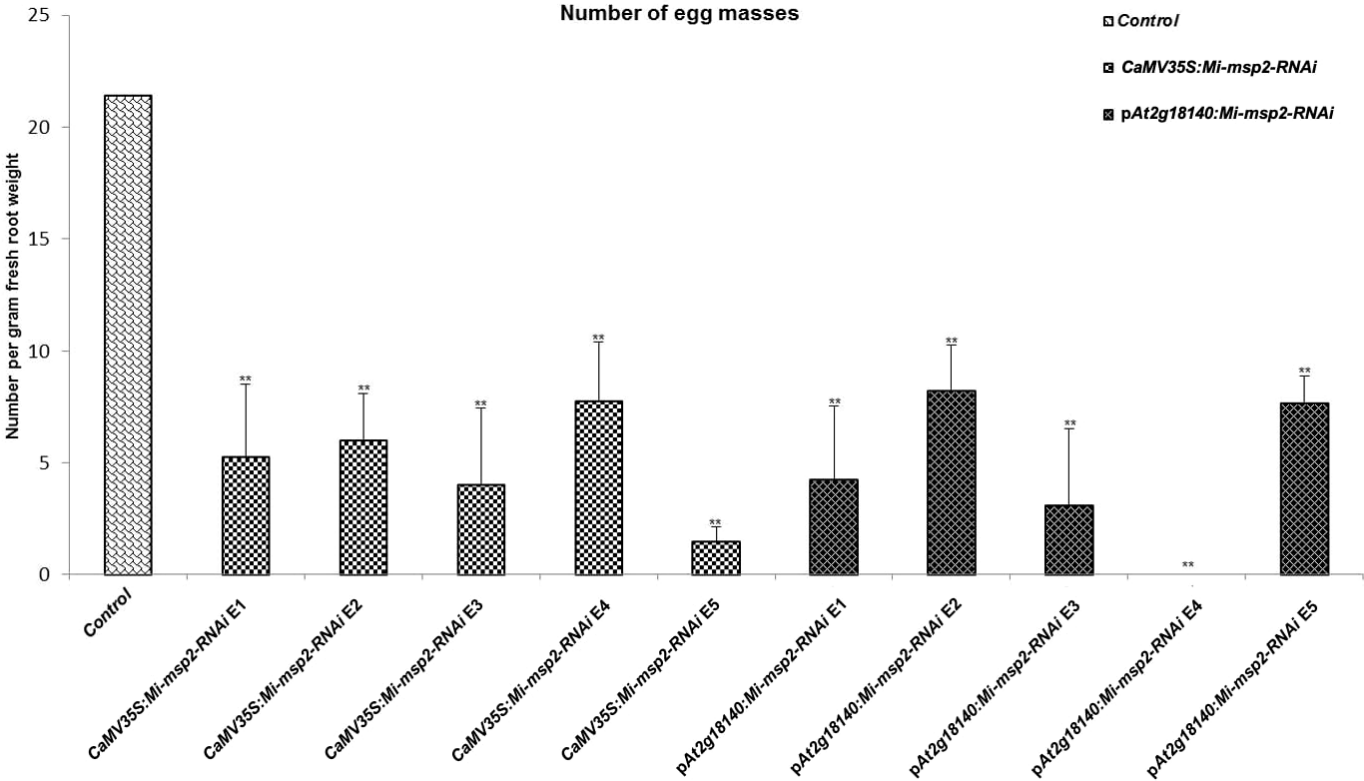
Five independent RNAi lines each for constructs *CaMV35S:Mi-msp2* and p*At2g18140::Mi-msp2-RNAi* showing a reduction in infection represented by a reduction in the number of egg masses produced by the females in infected roots compared to the control line evaluated for *M. incognita* infection. Each bar represents the mean ± SE. Double asterisks indicate statistically significant differences by one-way ANOVA and Tukey’s test (p ≤ 0.01).

**Figure 5:**
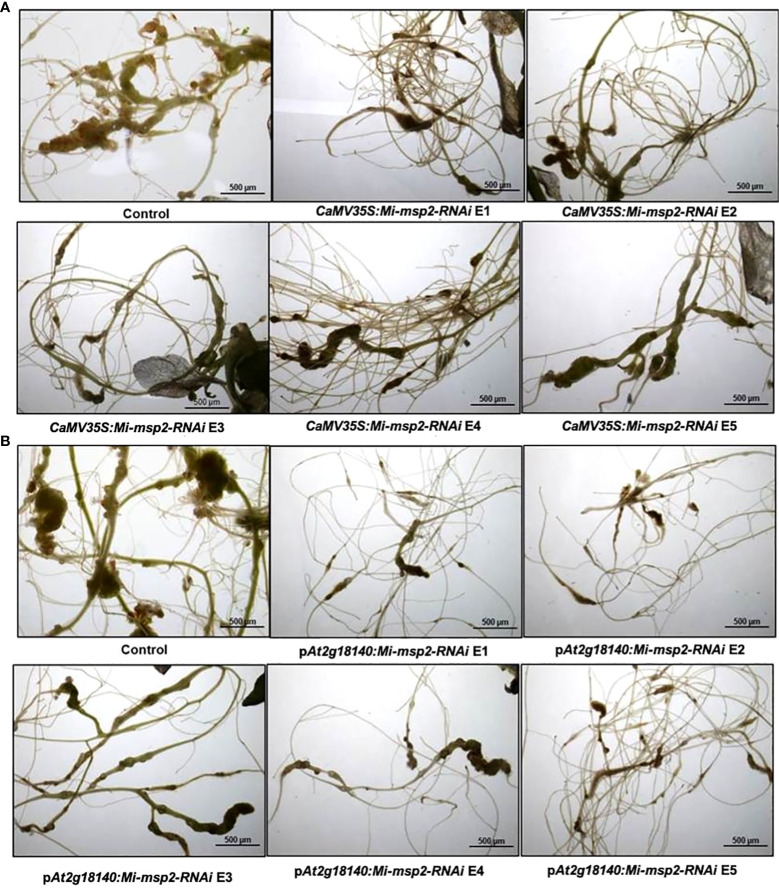
Representative images of *M. incognita*-infected *Arabidopsis thaliana* roots of control and RNAi plants. **(A)** Roots of a control plant compared with *CaMV35S::Mi-msp2-RNAi* RNAi lines (*CaMV35S::Mi-msp2-RNAi* E1, *CaMV35S::Mi-msp2-RNAi* E2, *CaMV35S::Mi-msp2-RNAi* E3, *CaMV35S::Mi-msp2-RNAi* E4 and *CaMV35S::Mi-msp2-RNAi* E5) and **(B)** Roots of a control plant compared with p*At2g18140::Mi-msp2-RNAi RNAi* lines (p*At2g18140::Mi-msp2-RNAi* E1, p*At2g18140::Mi-msp2-RNAi* E2, p*At2g18140::Mi-msp2-RNAi* E3, p*At2g18140::Mi-msp2-RNAi* E4 and p*At2g18140::Mi-msp2-RNAi* E5).

Five independent homozygous T_3_ transgenic events (E1, E2, E3, E4 and E5) of both promoter-driven *Mi-msp2* RNAi lines, namely, *CaMV35S::Mi-msp2-RNAi* and p*At2g18140::Mi-msp2-RNAi*, were selected for *M. incognita* infection analyses. The intensity of infection of the RNAi lines (of both promoters) was compared to the control plants (wild-type *Col-0*). The recorded numbers of galls, females and egg masses per gram fresh weight of root were compared (**Figures 2**–**4**). All of the transgenic events of the *Mi-msp2* RNAi lines (*CaMV35S::Mi-msp2-RNAi* and p*At2g18140::Mi-msp2-RNAi*) depicted a significant reduction in *M. incognita* infection compared to the control plants, indicating successful gene silencing. For the *CaMV35S::Mi-msp2-RNAi* lines, the percent reduction in the numbers of galls was recorded as 56%, 61%, 54%, 57% and 52% for *CaMV35S::Mi-msp2-RNAi* E1, *CaMV35S::Mi-msp2-RNAi* E2, *CaMV35S::Mi-msp2-RNAi* E3, *CaMV35S::Mi-msp2-RNAi* E4 and *CaMV35S::Mi-msp2-RNAi* E5, respectively (**Figure 2**). The reduction in the number of females was 60%, 64%, 61%, 59% and 66% for *CaMV35S::Mi-msp2-RNAi* E1, *CaMV35S::Mi-msp2-RNAi* E2, *CaMV35S::Mi-msp2-RNAi* E3, *CaMV35S::Mi-msp2-RNAi* E4 and *CaMV35S::Mi-msp2-RNAi* E5, respectively (**Figure 3**). The reduction in the number of egg masses was the maximum recorded as 83%, 80%, 87%, 75% and 95% for *CaMV35S::Mi-msp2-RNAi* E1, *CaMV35S::Mi-msp2-RNAi* E2, *CaMV35S::Mi-msp2-RNAi* E3, *CaMV35S::Mi-msp2-RNAi* E4 and *CaMV35S::Mi-msp2-RNAi* E5, respectively (**Figure 4**). In the p*At2g18140::Mi-msp2-RNAi* transgenic lines, the number of galls were reduced by 62%, 59%, 63%, 63% and 56% for p*At2g18140::Mi-msp2-RNAi* E1, p*At2g18140::Mi-msp2-RNAi* E2, p*At2g18140::Mi-msp2-RNAi* E3, p*At2g18140::Mi-msp2-RNAi* E4 and p*At2g18140::Mi-msp2-RNAi* E5, respectively (**Figure 2**). The reduction in the number of females was 64%, 66%, 68%, 67% and 64% for p*At2g18140::Mi-msp2-RNAi* E1, p*At2g18140::Mi-msp2-RNAi* E2, p*At2g18140::Mi-msp2-RNAi* E3, p*At2g18140::Mi-msp2-RNAi* E4 and p*At2g18140::Mi-msp2-RNAi* E5, respectively (**Figure 3**). Similarly, the reduction in the number of egg masses was the maximum recorded as 86%, 73%, 89%, 100% and 75% for p*At2g18140::Mi-msp2-RNAi* E1, p*At2g18140::Mi-msp2-RNAi* E2, p*At2g18140::Mi-msp2-RNAi* E3, p*At2g18140::Mi-msp2-RNAi* E4 and p*At2g18140::Mi-msp2-RNAi* E5, respectively (**Figure 4**). A significant decline in infection with constitutive promoters and the gall specific promoter-driven RNAi lines for the secretory gene *Mi-msp2* reveals the potential applicability of the gall specific promoter p*At2g18140* in nematode control (**Figure 5**).

### 4.3 Comparative analyses of morphometry of adult nematode females feeding on infected transgenic *Arabidopsis* RNAi lines *CaMV35S::Mi-msp2-RNAi* and p*At2g18140::Mi-msp2-RNAi* and control plants

**Figure 6:**
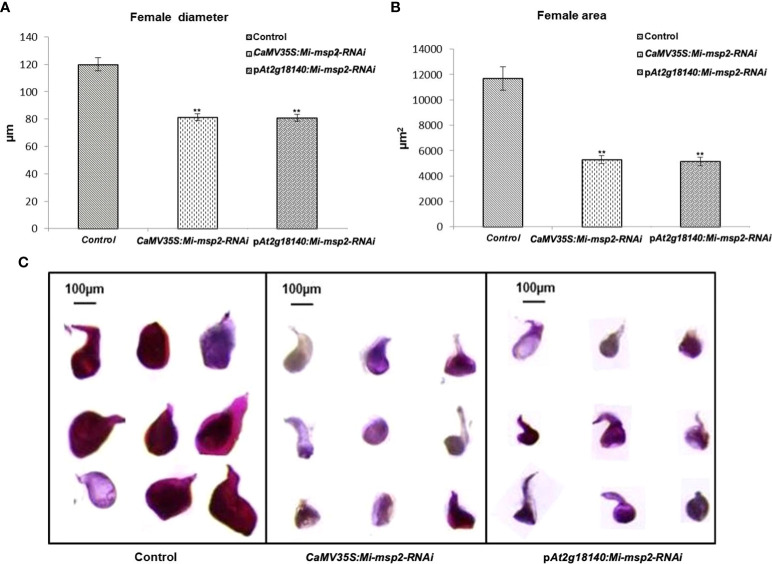
Morphological size comparison of female nematodes isolated from the control and RNAi plant lines *CaMV35S::Mi-msp2-RNAi* and p*At2g18140::Mi-msp2-RNA*i (cumulative females from all five lines of each of the RNAi constructs). **(A)** Average female width (μm), **(B)** average female area (μm^2^). Each bar represents the mean ± SE. Double asterisks indicate statistically significant differences according to one-way ANOVA and Tukey’s test (p ≤ 0.01). Representative images of adult females isolated from **(C)** Control, *CaMV35S::Mi-msp2-RNAi* and *pAt2g18140::Mi-msp2-RNAi* RNAi lines.

For the assessment of any differences in the growth and development of adult female nematodes, the sizes of the females isolated from the dsRNA-expressing transgenic lines (*CaMV35S::Mi-msp2-RNAi* and p*At2g18140::Mi-msp2-RNAi*) and control plants were compared. Morphometric analyses were conducted to assess the effect of silencing on the growth patterns by measuring the average surface area (μm^2^) and the average width (μm) of the feeding females. Twenty females were selected from each of the five RNAi lines of *CaMV35S::Mi-msp2-RNAi* and p*At2g18140::Mi-msp2-RNAi* and also from the control plants. Their lengths and diameters were documented and averaged. A visible reduction in size along with slight deformities in shape were evident in the cases of females feeding on transgenic RNAi lines compared to those feeding on control plants (**Figure 6C**). The reduction in the average diameter for females isolated from the *CaMV35S::Mi-msp2-RNAi* and p*At2g18140::Mi-msp2-RNAi* lines was 32% in both cases (**Figure 6A**). The reduction in the average area for females isolated from the *CaMV35S::Mi-msp2-RNAi* lines was recorded as 55% and for the p*At2g18140::Mi-msp2-RNAi* lines as 56% compared to the females isolated from the control plants (**Figure 6B**). In previous studies the reduction in female width and area for *Mi-msp2* RNAi lines was reported to be 32.2% and 54.8% respectively (Joshi et al., 2019). These results indicate that the *Mi-msp2* gene driven by both promoters affected the development of feeding *M. incognita* females.

### 4.4 Fecundity of *M. incognita* females feeding on *CaMV35S::Mi-msp2-RNAi* line and *pAt2g18140::Mi-msp2-RNAi* line plants and control plants

**Figure 7:**
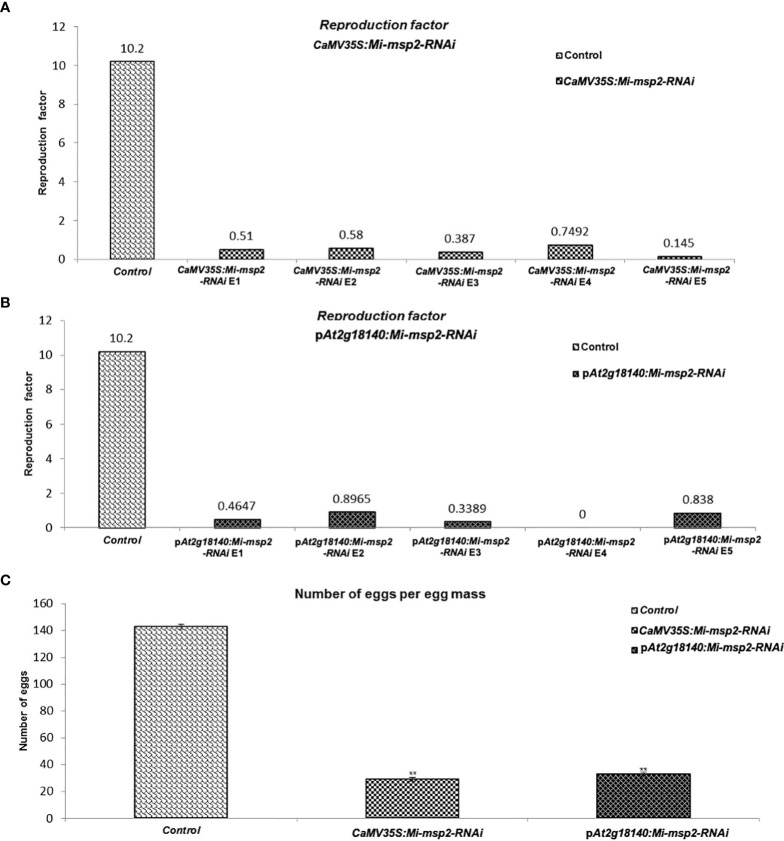
Fecundity of *M*. *incognita*. **(A)** Reproductive factors for the control and *CaMV35S::Mi-msp2.* **(B)** Reproductive factors for the control and p*At2g18140::Mi-msp2-RNAi.* **(C)** The average number of eggs per egg mass isolated from the control and *CaMV35S::Mi-msp2-RNAi* and p*At2g18140::Mi-msp2-RNAi* (cumulative egg masses from five lines of each RNAi construct). Each bar represents the mean ± SE. Double asterisks indicate statistically significant differences according to one-way ANOVA and Tukey’s test (p ≤ 0.01).

The reduction in the number of eggs was recorded as 80% for *CaMV35S::Mi-msp2-RNAi* and 77% for p*At2g18140::Mi-msp2-RNAi* (**Figure 7C**). Thus, silencing of the gene *Mi-msp2* driven by the promoters *CaMV35S* and p*At2g18140* successfully hampered the fecundity of *M. incognita*.

Another method to estimate nematode fecundity was the reproduction factor, which was calculated by measuring the number of juveniles hatched from the cumulative egg masses isolated from five independent RNAi lines of both *CaMV35S::Mi-msp2-RNAi* and p*At2g18140::Mi-msp2-RNAi*. For the egg masses excised from the five independent RNAi lines of *CaMV35S*::*Mi-msp2*, the reduction in the reproduction factor was 0.145–0.749 compared to 10.2 for the control plant juveniles (**Figure 7A**). The reproduction factor for the five independent RNAi lines of p*At2g18140::Mi-msp2-RNAi* was decreased to 0–0.896 compared to 10.2 for the control plant juveniles (**Figure 7B**). Comparable results were obtained in previous reports for the effector gene *Mi-msp2* RNAi lines (Joshi et al., 2019).

### 4.5 Gene expression analysis for *Mi-msp2* in females feeding on *CaMV35S::Mi-msp2-RNAi* and *pAt2g18140::Mi-msp2-RNAi* lines

**Figure 8:**
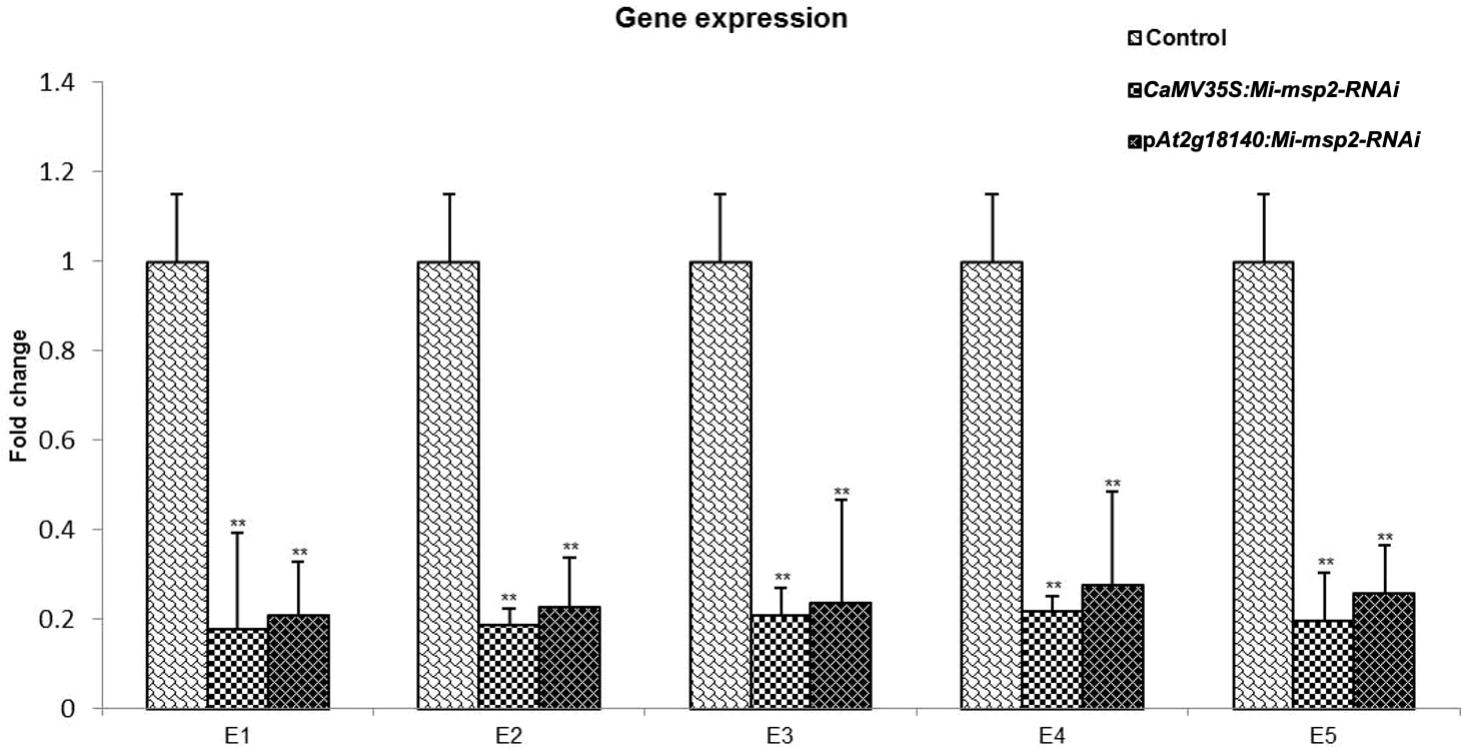
qRT–PCR-based expression analysis of *M. incognita* females isolated from infected roots of the control plants and five independent lines of each RNAi construct *CaMV35S::Mi-msp2-RNAi* and p*At2g18140::Mi-msp2-RNAi*. Each bar represents the mean ± SE. Double asterisks indicate statistically significant differences according to one-way ANOVA based on F and P values (p ≤ 0.01).

The expression of the *Mi-msp2* gene was observed to be downregulated relative to the nematode *actin* gene in each RNAi line. The reduction in gene expression for the *CaMV35S::Mi-msp2-RNAi* RNAi lines was 82%, 81%, 79%, 78% and 80% for E1, E2, E3, E4 and E5, respectively, compared to the control (**Figure 8**). The reduction in gene expression for the p*At2g18140::Mi-msp2-RNAi* lines was 79%, 77%, 76%, 72% and 74% for E1, E2, E3, E4 and E5, respectively, compared to the control (**Figure 8**). Thus, from the reduced transcript levels, we can conclude that dsRNA for the effector gene *Mi-msp2* was efficiently expressed by the RNAi constructs under the control of both promoters CaMV35S and p*At2g18140* and that HD-RNAi-based silencing was successful for the targeted gene.

## 5 Discussion

The NRRS promoters are responsible for triggering gene expression under the external stimulus provided by the pathogen at the time of nematode infection. The expression of the *Arabidopsis* promoter p*At2g18140* is highly restricted to the RKN galls present in the infected plant roots where the sedentary nematodes are at their feeding sites among the giant cells (Kakrana et al., 2017). When subjected to *M. incognita* infection, expression from this promoter was found to be spatiotemporally localized and it was not expressed in any other plant tissue or organ in the transgenic plants. This promoter is specifically expressed in the roots of infected plants as visualized by GUS expression (Kakrana et al., 2017).

The *At2g18140* gene encodes a plant peroxidase protein that has a plausible function in host-plant parasitic nematode interactions (Vercauteren et al., 2001; Jammes et al., 2005; Severino et al., 2012). Previously, plant peroxidases were found to assist in host plant parasitization in *Coffea canephora* sp., hence confirming the conserved roles of peroxidases in response to PPNs (Severino et al., 2012). Peroxidases are functional in the hypodermis and enable scavenging of host-derived reactive oxygen species, thereby protecting the external cell membranes from oxidation.

As one of the most widespread plant parasites, PPNs are a constant threat to many crops. Able to infect almost all of the vascular plants, they pose a threat to agriculture worldwide (Sato et al., 2019). The available genomic data and molecular information for PPNs can enable the development of methodologies to curb nematode infestation. RNAi technology has provided a promising way to curb nematode infection by silencing targeted nematode genes (Fire et al., 1998). Indigenous to *M. incognita*, the *Mi-msp* effector genes, a class of key genes involved in nematode parasitism, have served as targets for effective resistance *via* HD-RNAi. Silencing of various effector genes has directly affected the functionality of nematode parasitism to different degrees, as reported in the cases for effectors *Misp12*, *Mimsp40*, *Mimsp18*, *Mimsp20*, *Mi-msp-1*, *Mi-msp2*, *Mi-msp3*, *Mi-msp5* and *Mi-msp24* (Xie et al., 2016; Niu et al., 2016; Shivakumara et al., 2017; Chaudhary et al., 2019; Joshi et al., 2019; Joshi et al., 2020). The fusion constructs for the effector genes *Mi-msp1*, *Mi-msp16*, *Mi-msp20* and *Mi-cpl*, *Mi-icl*, *Mi-sf* have been successfully transformed in crop plants like eggplant and tomato to impart gene silencing *via* HD-RNAi (Hada et al., 2021; Lisei-de-Sá et al., 2021).

The expression of RNAi constructs under the control of their constitutive promoters serves the purpose of gene silencing, but a controlled targeting approach using the NRRS promoter will be more specific while effectively deterring PPN infection (Goddijn et al., 1993; Kakrana et al., 2017). Over the past few decades, various NRRS promoters have been found to be effectively activated in response to PPN infection, such as *TobRB7*, *LEMMI9*, *Hahsp17*.*7G4*, *AtCel-1*, *AtWRKY23*, *ZmRCP*-1, p*At1g74770* and p*At2g18140* (Opperman et al., 1994; Escobar et al., 1999; Escobar et al., 2003; Sukno et al., 2006; Grunewald et al., 2008; Onyango et al., 2016; Kakrana et al., 2017). These NRRS promoters can be employed to drive RNAi constructs for specific nematode target genes to dissuade PPN infection (Sukno et al., 2006; Kakrana et al., 2017). TobRB7 was used to drive dsRNA against the *MjTis11* gene of *M. javanica* in tobacco, which resulted in a nonsignificant reduction in the number of galls. The probable reason for this could be the weak promoter nature of TobRB7. A significant reduction of 32% was observed in dsRNA expressing RNAi lines from the *M. incognita* splicing gene under the control of the NRRS promoter p*At1g74770* (Kakrana et al., 2017).

The class *Mi-msp* of nematode effector genes has been observed to impart promising results to curb PPN infection when employed *via* HD-RNAi technology (Niu et al., 2016; Joshi et al., 2019; Joshi et al., 2020). The effector gene *Mi-msp2* originates in the subventral oesophageal glands of *M. incognita* and is maximally expressed in the earlier stages of the lifecycle (Joshi et al., 2019). Recently the HD-RNAi based silencing of the genes involved in chitin biosynthesis has been demonstrated in tobacco lines. The reduction in *M. incognita* infection has been reported in terms of number of eggs and root knots of up to 90% (Mani et al., 2020). This suggests its involvement in penetration and migration in host plants. The *Mi-msp2* gene has shown a promising reduction in *M. incognita* infection in *Arabidopsis* transgenic RNAi lines. The reduction in infection in terms of the number of galls for the constitutive promoter RNAi line *CaMV35S::Mi-msp2-RNAi* was similar to that of the gall specific promoter RNAi line p*At2g18140::Mi-msp2-RNAi.* In a similar manner, all of the lines of both constructs showed a reduction in the number of females, which was comparable for the RNAi lines driven by both promoters. The development of J2s feeding on the *CaMV35S::Mi-msp2-RNAi* and p*At2g18140::Mi-msp2-RNAi* lines was hampered, as demonstrated by the reduction in the diameter and area in the extracted fully developed adult females from all of the RNAi lines compared to the control plants. There was a reduction in both reproduction factor and the number of eggs per egg mass in *CaMV35S::Mi-msp2-RNAi* and p*At2g18140::Mi-msp2-RNAi* RNAi lines with a collective reduction in fecundity of the next generation of *M. incognita*. The females feeding on the RNAi lines also presented reduced production of *Mi-msp2* transcripts compared to females feeding on control plants.

## 6 Conclusion

Expressing defence-related pathogen genes under the control of constitutive promoters has been observed to cause the development of disease symptoms even in the absence of respective pathogens, hampering the growth of transgenic plants, resulting in sterile transgenic plants, and causing inefficient expression of the genes of interest and their expression in unrelated tissues (FitzGerald et al., 2004; Venter, 2007). Naturally occurring constitutive promoters might underperform and have downregulated expression at the desired site, as seen for the promoter *Act2* (of *A. thaliana*) in seed coats and the actin promoter (of rice) in xylem tissue (McElroy et al., 1990; An et al., 1996; Biłas et al., 2016). To overcome these problems while providing nematode resistance, NRRS promoters can serve as a tool for quantitatively, temporally and spatially controlling the expression of a dsRNA construct transferred *via* HD-RNAi. Here, we constructed a promoter and an effector gene RNAi system that allows for the activation of effective silencing of the gene of interest only after the host is attacked by the nematode. The peroxidase promoter p*At2g18140* can be applied for producing localized dsRNA constructs of various PPN parasitism genes at the time of infection in several host plants. Our results show that in a controlled manner, suppression by RNAi could be achieved by selecting a suitable selective promoter instead of a constitutive promoter. The tissue-specific HD-RNAi suppression technique has also proven to be a useful tool for the production of transgenic crop plant materials, providing insights into fundamental studies of biotic stress/disease resistance. Temporal and spatial gene control as an alternative approach *via* inducible promoters is desirable as a new system for cases with external stimulus-specific responses. Applying specifically inducible silencing systems can overcome lethality problems and aid in revealing information about gene function in specific knockout studies. We have developed a nematode-inducible system offering rigorous, noninvasive, stimuli-controlled gene expression in plants, a technology that can be further expanded to various crop systems. This system can be applied to generate a series of effective silencing constructs *via* a gene pyramiding technique, clubbing together different RNAi constructs expressed by the gall specific promoter p*At2g18140*.

## Data availability statement

The original contributions presented in the study are included in the article/**Supplementary Material**. Further inquiries can be directed to the corresponding author.

## Author contributions

PJ conceived the idea. PJ, AC, RB, and AS designed the experiment. IJ, AK, and DK conducted the experiments. IJ performed the data analyses. IJ and DK prepared the Figures and/or tables. IJ wrote the manuscript. PJ, AC, RB, and AS edited the manuscript. All authors contributed to the article and approved the submitted version.

## Funding

IJ is also thankful to the University Grants Commission, India, for providing funding in the form of the UGC-JRF fellowship (2013-2018). The present study was also supported by a grantfrom the ICAR-NASF (formerly known as NFBSFARA) (code:NFBSFARA/RNA-3022) project during the years 2012-2015.

## Acknowledgments

The authors would like to gratefully thank the staff of the National Phytotron Facility (NPF), IARI, New Delhi, India for providing space to conduct experiments on *Arabidopsis* plants.

## Conflict of interest

The authors declare that the research was conducted in the absence of any commercial or financial relationships that could be construed as a potential conflict of interest.

## Publisher’s note

All claims expressed in this article are solely those of the authors and do not necessarily represent those of their affiliated organizations, or those of the publisher, the editors and the reviewers. Any product that may be evaluated in this article, or claim that may be made by its manufacturer, is not guaranteed or endorsed by the publisher.
