# Supplementary material for: Gall-specific promoter, an alternative to the constitutive CaMV35S promoter, drives host-derived RNA interference targeting Mi-msp2 gene to confer effective nematode resistance
Source: Front Plant Sci. 2022 Nov 8;13:1007322. doi: 10.3389/fpls.2022.1007322 (PMC9679145; doi:10.3389/fpls.2022.1007322)
Supplement: Supplementary file 1 [file DataSheet_1.docx]

**Gall-specific promoter, an alternative to the constitutive *CaMV35S* promoter, drives host-derived RNA interference targeting *Mi-msp2* gene to confer effective nematode resistance**

Ila Joshi^1,2^, Anil Kumar^1,3^, Deshika Kohli^1^, Ramcharan Bhattacharya^1^, Anil Sirohi^4^, Ashok Chaudhury^2^ and Pradeep K. Jain^1^

^1^ICAR-National Institute for Plant Biotechnology, Pusa Campus, New Delhi 110012.

^2^Department of Bio and Nano Technology, Bio & Nano Technology Centre, Guru Jambheshwar University of Science and Technology, Hisar-125001, Haryana, India.

^3^Department of Entomology, Nematology and Chemistry Units, Agricultural Research Organization, The Volcani Center, Bet Dagan, 5025004, Israel

^4^Division of Nematology, ICAR-Indian Agricultural Research Institute, New Delhi 110012, India.

RUNNING TITLE- Gall-specific promoter for RKN resistance *via* HD-RNAi.

Supplementary table S1: List of primers used in the study.

| Gene primer | Sequence (5´-3´) | Restriction enzyme | Amplicon size bp |
| --- | --- | --- | --- |
| Primers for RNAi construct preparation | | | |
| p*At2g18140* F_*Mlu*I | TCGACACGCGTGGGTTGGACATTAGGATAGGG | *Mlu*I | 1572 |
| p*At2g18140* R_*Xho*I | TCGACC/TCGAGTTGTCGCTAGCTAGATCAAGAA | *Xho*I |  |
| *Mi-msp2* F sense *Eco*RI | TAGCAG/AATTCCCAATCGGGTTTATTTGGGCT | *Eco*RI | 590 |
| *Mi-msp2* R sense *Kpn*I | TTGACGGTAC/CGCATGAATCTTAACTTTCGGA | *Kpn*I |  |
| *Mi-msp2* F antisense *Hind*III | TATTAA/AGCTTGCATGAATCTTAACTTTCGGA | *Hind*III | 590 |
| *Mi-msp2* R antisense *Xba*I | CTAAGT/CTAGACCAATCGGGTTTATTTGGGCT | *Xba*I |  |
| PDK p*HANNIBAL*_F | TTTTGTTAGAAATTCCAATCTGC | -- | 154 |
| PDK p*HANNIBAL*_R | TGATAGATCTTGCGCTTTGTT |  |  |
| Primers for gateway cloning | | | |
| *Mi-msp2* GW F | tctcccgggCCAATCGGGTTTATTTGGGCT | -- | 587 |
| *Mi-msp2* GW R | ctcccgggGCATGAATCTTAACTTTCGGA | -- |  |
| Primers for Quantitative Real Time PCR | | | |
| Mi-msp2 RT 1_F | GTTACTTCGGCAACCTCAAA | -- | 150 |
| Mi-msp2 RT 1_R | CTTTGCGTCTTTGAATTCGT | -- |  |
| Act-N1_F QRT | TACGCCAACACTGTCCTTTC | -- | 125 |
| Act-N1_R QRT | CGCTCAGGAGGTGCAATAAT | -- |  |
| Mi 18S QRT F 1 | GGCTCATGGTGGAAAGTATG | -- | 167 |
| Mi 18S QRT R 1 | CCCCAGTGTAATGTCCTTTG | -- |  |
| Primers for secretory gene | | | |
| Mi-msp2 F | tctcccgggCCAATCGGGTTTATTTGGGCT | -- | 587 |
| Mi-msp2 R | tctcccgggGCATGAATCTTAACTTTCGGA | -- |  |


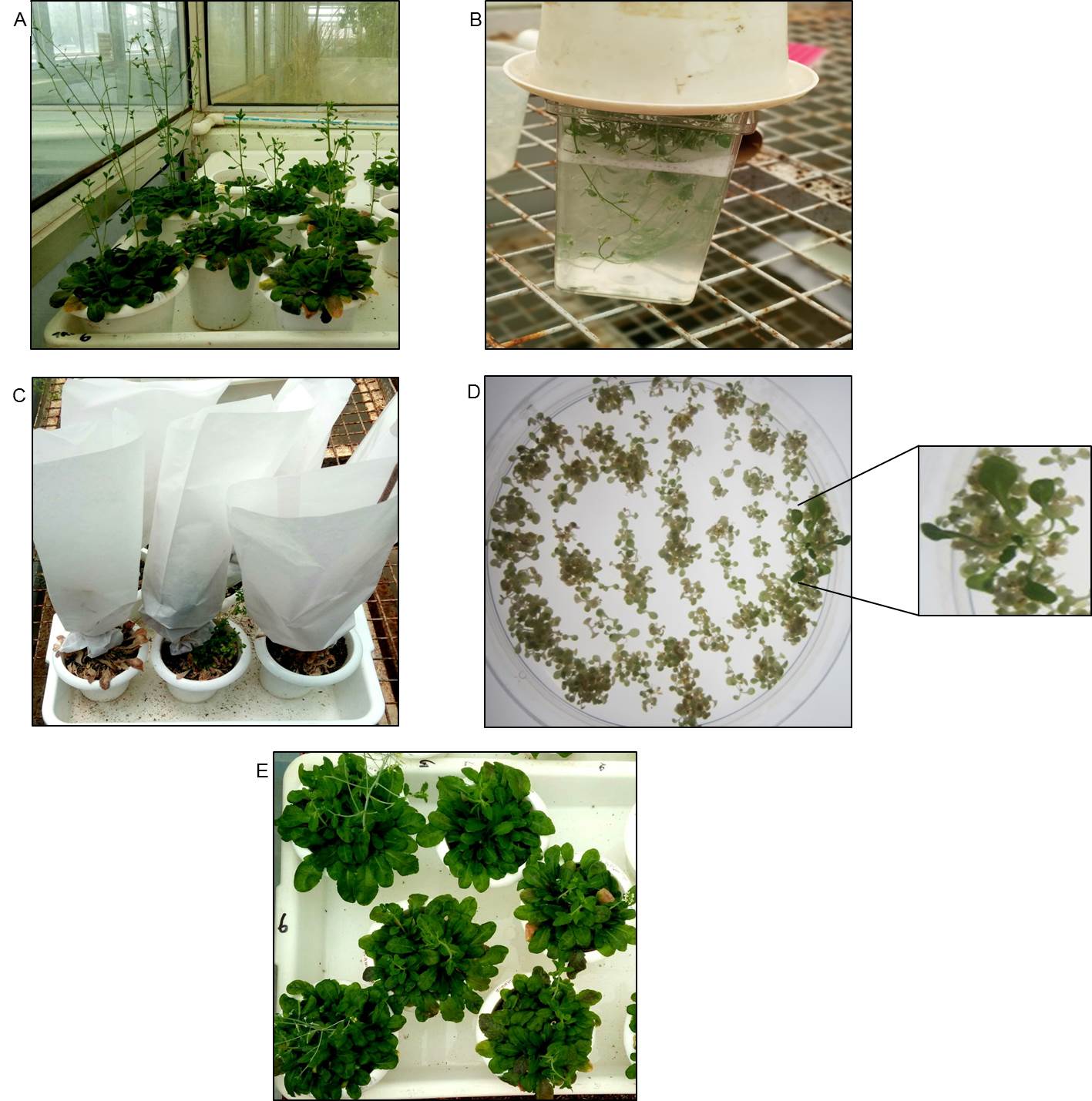


Supplementary Fig. S1. Generation of p*At2g18140::Mi-msp2-RNAi*  transgenic lines *via* floral dip method. (A) Propagation of wild-type *Col-0* plants. (B) Floral dip of *Arabidopsis* buds in the *A. tumefaciens* culture with p*At2g18140::Mi-msp2-RNAi*  construct. (C) Bagging of the plants for seed collection. (D) Representative image for confirmation of p*At2g18140::Mi-msp2-RNAi* transgenic plants on hygromycin selective MS media. (E) Further propagation of selected transgenic lines to T_3_ generation.


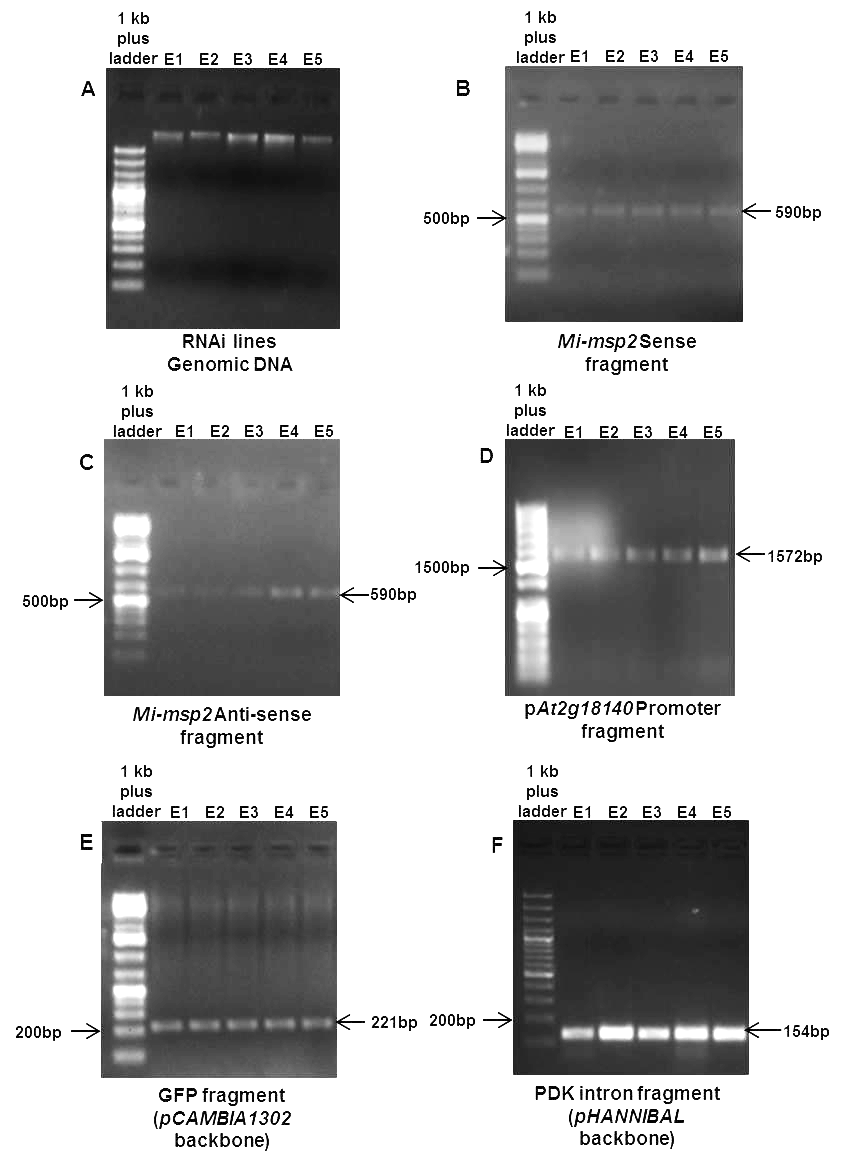


Supplementary Fig. S2. PCR confirmation of five T_3_ transgenic lines for p*At2g18140::Mi-msp2-RNAi* (p*At2g18140::Mi-msp2-RNAi* E1, p*At2g18140::Mi-msp2-RNAi* E2, p*At2g18140::Mi-msp2-RNAi*  E3, p*At2g18140::Mi-msp2-RNAi* E4 and p*At2g18140::Mi-msp2-RNAi* E5) insert fragments. (A) Genomic DNA of five transgenic RNAi lines, (B) PCR amplified *Mi-msp2* gene sense fragment (590 bp), (C) PCR amplified *Mi-msp2* gene anti-sense fragment (590 bp),(D) PCR amplified p*At2g18140* promoter fragment (1572 bp), (E) PCR amplified GFP fragment from the *pCAMBIA 1302* backbone (221 bp) and (F) PCR amplified PDK intron fragment from the *pHANNIBAL* backbone (154bp).
